# Blood Volume Status in ME/CFS Correlates With the Presence or Absence of Orthostatic Symptoms: Preliminary Results

**DOI:** 10.3389/fped.2018.00352

**Published:** 2018-11-15

**Authors:** C. (Linda) M. C. van Campen, Peter C. Rowe, Frans C. Visser

**Affiliations:** ^1^Stichting Cardiozorg, Hoofddorp, Netherlands; ^2^Department of Pediatrics, Johns Hopkins University School of Medicine, Baltimore, MD, United States

**Keywords:** orthostatic intolerance, chronic fatigue syndrome, myalgic encephalomyelitis, blood volume, POTS, dual isotope scan

## Abstract

**Introduction:** Conflicting data have been published on the reduction of circulating blood volume in adults with Myalgic encephalomyelitis/chronic fatigue syndrome (ME/CFS). The aim of the present study was to compare blood volumes based on the presence or absence of orthostatic symptoms.

**Methods and results:** Twenty consecutive adults with ME/CFS participated in the study. All underwent dual isotope blood volume measurement and were evaluated for a clinical suspicion of orthostatic intolerance (OI). The mean age was 34 (10) years, and median duration of disease was 7.5 (6–10) years. The mean (SD) absolute blood volume was 59 (8) ml/kg, a value −11 (7) ml/kg below the reference blood volume. Of the 12 patients, 4 had no OI and 8 had a clinical suspicion of OI. In 8 patients with OI, absolute blood volumes were significantly lower than for the 4 without OI (56 [2] vs. 66 [5]; *p* < 0.05) as were the differences between the measured and the reference blood volume (−14 [2]; vs. −4 [3]; *p* < 0.02).

**Conclusions:** Adults with ME/CFS had a significantly lower blood volume if they had a clinical suspicion of OI compared to those without a clinical suspicion of OI, as well as a significantly lower blood volume compared to the expected value. The data suggest that accounting for symptoms of OI could enhance the detection of the subset with reduced blood volume.

## Introduction

Myalgic encephalomyelitis/chronic fatigue syndrome (ME/CFS) is characterized by a persistent severe fatigue, diminished exercise tolerance, post exertional malaise, unrefreshing sleep, and impaired memory and concentration. A prominent feature is also dizziness and/or lightheadedness. More individuals with ME/CFS than healthy controls experience lightheadedness and syncope (1–3). Studies have shown conflicting results regarding circulating blood volume in ME/CFS patients compared to a healthy population (4–6). Lin et al. (7) identified a relation between red blood cell (RBC) volume deficiency and the presence of orthostatic intolerance (OI) in chronic OI patients and especially in those with postural orthostatic tachycardia syndrome (POTS). To explore whether this observation of OI in a non-ME/CFS subject group might also be applicable in a population with ME/CFS, the aim of the present study was to compare measured blood volume in adults with ME/CFS after sub-grouping by the presence or absence of OI symptoms.

## Materials and methods

Individuals with ME/CFS were eligible for this study if they were being evaluated at the Stichting CardioZorg, a cardiology clinic with a special interest in ME/CFS. ME/CFS was considered present if participants met both the CFS (8) and the ME criteria (9) with no other major comorbidities.

Beginning in 2010, as part of routine care, we began measuring blood volume using the standard dual isotope erythrocyte labeling technique (${\text{Na}}_{2}^{51}$CrO_4_ and ^125^I-human serum albumin) (10) at the department of Nuclear Medicine of the Free University Hospital Amsterdam. Blood volume was compared with the ideal weight of patients (11), using the method of Devine (12). Due to the high cost of the blood volume measurements (3500 USD) and the loss of funding for these studies, patient enrollment was limited to the first 12 individuals.

The presence or absence of a clinical suspicion of OI was based on the history taken by an experienced cardiologist (FCV) who asked how individuals felt in the following circumstances: while waiting in line, at receptions, while shopping, while sitting still for long periods, and when exposed to warm/stressful circumstances (e.g., summer weather, after hot showers, after episodes of fear or pain), (13–15). Those with increased lightheadedness and other symptoms in these settings were considered to have a clinical suspicion of OI. The use of clinical data for descriptive studies was approved by the ethics committee of the Slotervaart Hospital.

Scores were tested for normal distribution using the Shapiro Wilk test in SPSS (IBM SPSS version 21). Normally distributed data were presented as mean (SD), and data that were not normally distributed were presented as a median (IQR). Data were compared with the student' *t*-test for unpaired data where appropriate. A *p*-value < 0.05 was considered significantly different.

## Results

Among the 12 consecutive ME/CFS participants, the mean age was 34 (10) years, and the median duration of disease was 7.5 (6–10) years. In the total patient group the absolute blood volume was 59 (8) ml/kg. The reduction in blood volume from the reference standard based on ideal weight was −11(7) ml/kg. Based on the clinical history, 4 had no clinical suspicion of OI and 8 had a clinical suspicion of OI (two of whom had been diagnosed with POTS; in the remaining six, tilt table testing elsewhere had identified no hemodynamic abnormalities). Table 1 shows the baseline data and blood volumes in patients without (*n* = 4) and with (*n* = 8) OI. Those with OI were significantly younger. In those who reported symptoms of OI, the absolute blood volumes were significantly lower than in those without OI, as were the differences between measured blood volume and the reference blood volume.

**Table 1:** Demographic and volume characteristics of the study population^*^.

	**OI absent**	**OI present**	**p-value**
Female	100%	88%	ns
Height	173 (9) cm	170 (10) cm	ns
Weight	65 (9) kg	63 (13) kg	ns
Caucasian	100%	100%	ns
Age (years)	44 (4)	28 (9)	< 0.01
Median duration of CFS	9 (8–11) years	7 (5.8–10) years	ns

* *Unless otherwise specified, these values represent mean (SD. Diff, difference; OI, orthostatic intolerance)*.

**Figure 1:**
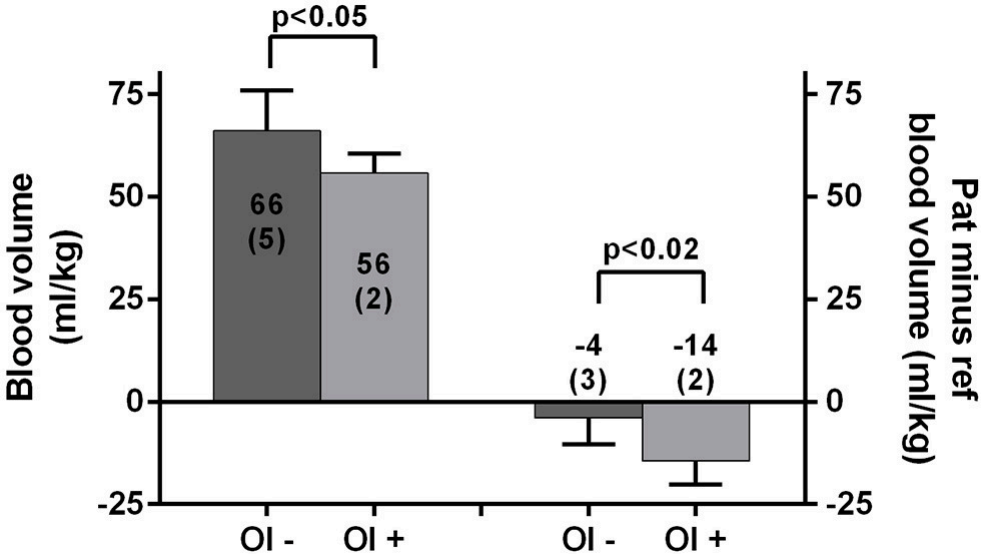
Absolute blood volumes (ml/kg) in those without and with OI are shown on the left y-axis. The differences between the measured blood volume minus the reference value (ml/kg) are shown on the right y-axis. Values are presented as mean (SD); OI– and OI+, clinical suspicion of orthostatic intolerance absent and present; Pat, patients; ref, reference.

Figure 1 shows the differences in blood volumes for patients with and without OI: 56 (2) vs. 66 (5) ml/kg; *p* < 0.05. The difference between the measured blood volume and the reference blood volume is shown in patients with and without OI: −14 (2) vs. −4 (3); *p* < 0.02. No significant correlation was found between the disease duration and absolute blood volume or the reduction in blood volume compared to the reference blood volume.

## Discussion

The main finding of this study is that blood volumes were significantly lower for adults with ME/CFS whose symptoms were consistent with orthostatic intolerance compared to those with no clinical suspicion of OI. The finding was present when we compared either absolute blood volume values or the percent reduction in blood volumes from the normal reference value for each individual. Our data are similar to a study in which only OI patients were investigated (7). Lin and colleagues compared the measured red blood cell (RBC) volume with an expected RBC volume, and found RBC volumes between 78% (POTS patients) and 85% (chronic OI patients without POTS) of the reference value.

Only a limited number of studies on blood volume have been performed in those with ME/CFS. Streeten and colleagues found in 12 female CFS patients that the RBC volumes were lower than that of female control subjects, but found in contrast that plasma and whole blood volumes were not significantly different from control subjects (6). Farquhar identified no significant difference in blood volume between ME/CFS patients and simultaneously studied age-matched controls (4), although there was a non-significant trend toward lower blood volume in those with ME/CFS. Hurwitz and colleagues examined 56 with ME/CFS (30 more severely affected and 26 non-severely affected). Total blood volume, erythrocyte volume, and plasma volume were not significantly different from 21 sedentary controls (5). However, when recalculating the reduction from ideal volumes, the percent total blood, plasma, and RBC volumes were all significantly lower in those with ME/CFS than in sedentary controls and also lower in those with severe ME/CFS compared to less severely affected individuals. Of interest, the mean absolute blood volume in our patient population (59 ml/kg) was mid-way between the values for those with severe ME/CFS (57 ml/kg) and non-severe ME/CFS (61 ml/kg) reported by Hurwitz et al. Newton et al. (16) found no significant difference for whole blood volumes between 41 with CFS and 10 healthy controls, but 68% of those with ME/CFS had a RBC volume below 95% of the expected mean volume for healthy individuals. Thirty-two percent had a normalized plasma volume below the lower limit of normal of 95%, suggesting a difference in the degree of reduction between plasma and RBC volumes. None of these studies classified participants according to the presence or absence of orthostatic intolerance.

The group with was younger than the group without orthostatic intolerance. Future studies will be able to determine whether this age difference persists in a larger sample. Of the limited number of studies regarding how blood volume varies over the lifespan in healthy volunteers, the data are consistent with either stable or declining blood volume with increasing age (17, 18) If the same relationship between blood volume and age is present for those with ME/CFS, then the older age of the group without orthostatic intolerance would have reduced the likelihood of detecting the difference we observed. Similarly, an age-matched group without orthostatic intolerance would be expected to increase the difference in blood volume between groups.

## Limitations

In the present study we did not include simultaneous control subjects. We acknowledge that the small sample size could have led to a type I statistical error. Thus, the data presented here should be considered preliminary and our results need to be confirmed in a larger study.

## Conclusion

This small study identified a lower absolute measured blood volume and a greater reduction in blood volume compared to expected normal values in those with ME/CFS who reported symptoms of orthostatic intolerance. The data suggest that accounting for orthostatic symptoms has the potential to better identify the subset of individuals with ME/CFS who have a reduced blood volume, which in turn would have implications for treatment and the prevention of disabling symptoms. Because a significant reduction of blood volume is an objectively demonstrable laboratory abnormality in ME/CFS patients, larger studies are warranted.

## Ethics statement

This trial was designed, conducted, and reported in accordance with the international Conference on Harmonization (ICH) Guidelines for Good Clinical Practice (GCP), applicable local regulations (including European Directive 2001/20/EC), and following the ethical principles laid down in the Declaration of Helsinki. The protocol was approved by the METC Slotervaartziekenhuis en Raede, Amsterdam, Netherlands.

## Data availability statement

The raw data supporting the conclusions of this manuscript will be made available by the authors, without undue reservation, to any qualified researcher.

## Author contributions

CvC, PR, and FV conceived the study. CvC and FV collected the data. CvC performed the primary data analysis and FV and PR performed secondary data analyses. All authors were involved in the drafting and review of the manuscript.

### Conflict of interest statement

The authors declare that the research was conducted in the absence of any commercial or financial relationships that could be construed as a potential conflict of interest.

## References

[1] Bou-Holaigah I Rowe PC Kan J Calkins H. The relationship between neurally mediated hypotension and the chronic fatigue syndrome JAMA (1995) 274:961–7. 7674527

[2] Rowe PC Bou-Holaigah I Kan JS Calkins H. Is neurally mediated hypotension an unrecognised cause of chronic fatigue? Lancet (1995) 345:623–4. 7898182 10.1016/s0140-6736(95)90525-1

[3] Ulas UH Chelimsky TC Chelimsky G Mandawat A McNeeley K Alshekhlee A. Comorbid health conditions in women with syncope. Clin Auton Res. (2010) 20:223–7. 10.1007/s10286-010-0070-x 20458514

[4] Farquhar WB Hunt BE Taylor JA Darling SE Freeman R. Blood volume and its relation to peak O(2) consumption and physical activity in patients with chronic fatigue. Am J Physiol Heart Circ Physiol. (2002) 282:H66–71. 10.1152/ajpheart.2002.282.1.H66 11748048

[5] Hurwitz BE Coryell VT Parker M Martin P Laperriere A Klimas NG. Chronic fatigue syndrome: illness severity, sedentary lifestyle, blood volume and evidence of diminished cardiac function. Clin Sci. (2010) 118:125–35. 10.1042/CS20090055 19469714

[6] Streeten DH Thomas D Bell DS. The roles of orthostatic hypotension, orthostatic tachycardia, and subnormal erythrocyte volume in the pathogenesis of the chronic fatigue syndrome. Am J Med Sci. (2000) 320:1–8. 10.1016/S0002-9629(15)40790-6 10910366

[7] Lin CJ Chu YK Chern CM. RBC volume deficiency in patients with excessive orthostatic decrease in cerebral blood flow velocity. J Chin Med Assoc. (2014) 77:174–8. 10.1016/j.jcma.2014.01.005 24612999

[8] Fukuda K Straus SE Hickie I Sharpe MC Dobbins JG Komaroff A. The chronic fatigue syndrome: a comprehensive approach to its definition and study. International Chronic Fatigue Syndrome Study Group. Ann Intern Med. (1994) 121:953–9. 7978722 10.7326/0003-4819-121-12-199412150-00009

[9] Carruthers BM van de Sande MI De Meirleir KL Klimas NG Broderick G Mitchell T. Myalgic encephalomyelitis: International Consensus Criteria. J Intern Med. (2011) 270:327–38. 10.1111/j.1365-2796.2011.02428.x 21777306 PMC3427890

[10] Fairbanks VF Klee GG Wiseman GA Hoyer JD Tefferi A Petitt RM. Measurement of blood volume and red cell mass: re-examination of 51Cr and 125I methods. Blood Cells Mol Dis. (1996) 22:169–86; discussion 186a–g. 10.1006/bcmd.1996.0024 8931957

[11] Feldschuh J Katz S. The importance of correct norms in blood volume measurement. Am J Med Sci. (2007) 334:41–6. 10.1097/MAJ.0b013e318063c707 17630591

[12] Devine BJ Gentamycin therapy drug. Intell Clin Pharm. (1974) 8:650–5.

[13] Grubb BP. Clinical practice. Neurocardiogenic syncope. N Engl J Med. (2005) 352:1004–10. 10.1056/NEJMcp042601 15758011

[14] Raj SR. Postural tachycardia syndrome (POTS) Circulation (2013) 127:2336–42. 10.1161/CIRCULATIONAHA.112.144501 23753844 PMC3756553

[15] Thieben MJ Sandroni P Sletten DM Benrud-Larson LM Fealey RD Vernino S. Postural orthostatic tachycardia syndrome: the Mayo clinic experience. Mayo Clin Proc. (2007) 82:308–13. 10.4065/82.3.308 17352367

[16] Newton JL Finkelmeyer A Petrides G Frith J Hodgson T Maclachlan L Reduced cardiac volumes in chronic fatigue syndrome associate with plasma volume but not length of disease: a cohort study. Open Heart (2016) 3:e000381 10.1136/openhrt-2015-000381 27403329 PMC4932290

[17] Davy KP Seals DR. Total blood volume in healthy young and older men. J Appl Physiol. (1985) (1994) 76:2059–62. 10.1152/jappl.1994.76.5.2059 8063668

[18] Jones PP Davy KP DeSouza CA van Pelt RE Seals DR. Absence of age-related decline in total blood volume in physically active females. Am J Physiol. (1997) 272:H2534–40. 10.1152/ajpheart.1997.272.6.H2534 9227528

